# Assessment of the impact of good pharmacy practices training among drug dispensers in Bangladesh

**DOI:** 10.3389/fphar.2023.1139632

**Published:** 2023-07-12

**Authors:** Sunjida Binta Ali, Nantu Chakma, Md. Saimul Islam, Raian Amzad, Md. lftakhar Hassan Khan, Md. Aziulla, Tanisha Momtaz, Abul Kalam Azad, Zaheer-Ud-Din Babar, Aliya Naheed

**Affiliations:** ^1^ Initiative for Non-Communicable Diseases, Health System and Population Studies Division, International Centre for Diarrhoeal Disease Research, Dhaka, Bangladesh; ^2^ Management Sciences for Health (MSH), Dhaka, Bangladesh; ^3^ Directorate General of Drug Administration (DGDA), Dhaka, Bangladesh; ^4^ Department of Pharmacy, BRAC University, Dhaka, Bangladesh; ^5^ Department of Pharmacy, University of Huddersfield, Huddersfield, United Kingdom

**Keywords:** drug dispensers, good pharmacy practice, grade C pharmacists, knowledge, practice, dispensing behavior

## Abstract

**Background:** Training improves dispensing behavior of drug dispensers in low- and middle-income countries. Between 2018 and 2020, a total of 5,059 Grade C pharmacists, who completed a 3-month training course for availing a “Grade C pharmacist certificate” were trained on Good Pharmacy Practice (GPP) in 11 districts in Bangladesh by Management Sciences for Health (MSH) under Better Health in Bangladesh (BHB) project. We assessed the impact of GPP among trained Grade C pharmacists under the BHB project compared to those who did not receive GPP training under the BHB project (non-trained), and explored the major challenges towards achieving GPP.

**Methods:** We created a database of trained Grade C pharmacists provided by MSH and randomly selected the trained Grade C pharmacists for recruitment following consent. We created another database of the non-trained Grade C pharmacist who were deployed within a 1-km radius of a trained Grade C pharmacist, and randomly recruited one non-trained against one trained Grade C pharmacist. A semi structured questionnaire was administered to obtain information about knowledge of GPP, including guidelines of dispensing medicines, temperature maintenance, medicine storage, counseling customers and labeling medicines. Dispensing behavior was directly observed following a structured tool. Chi-square test (for categorical variables) and independent sample t-tests (for continuous variables) were applied for comparison between the trained and the non-trained Grade C pharmacists. A logistic regression model was applied to explore an association between knowledge and practice between the two groups.

**Results:** Between February and March 2021, 220 trained and 220 non-trained Grade C pharmacists were recruited. Mean age (SD) of the participants was 41 years (10.5) and 98.4% were male. Compared to the non-trained, the trained Grade C pharmacists had better knowledge about the guidelines of dispensing medicines (97.7% vs 89.5%, *p* < 0.001), temperature maintenance (91.8% vs 45.5%, *p* = 0.001), medicine storage (92.3% vs 40.5%, *p* = 0.001) counseling customers (99.5% vs 92.3%, *p* < .001) and labeling medicines (91.0% vs 80%, *p* < 0.001). General dispensing behavior was observed to be better among the trained than the non-trained with labeling of medicines (63.2% vs 53.4%, *p* = 0.038), counseling customers (39.1% vs 28.6%, *p* = 0.021) and using a room thermometer for maintaining ambient temperature in the medicine shops (56.8% vs26.8%, *p* < 0.001). Bad behavior of the customers (39.5%) and lack of GPP knowledge among Grade C pharmacists (28.6%) were recognized to be challenges towards achieving GPP in Bangladesh.

**Conclusion:** Training led to better knowledge and practices about dispensing medicines among Grade C pharmacists in Bangladesh. Periodic training may promote achieving GPP in Bangladesh.

## 1 Introduction

Ensuring availability, affordability, accessibility, and appropriate use of quality medicines are essential for offering quality health services and preventing negative health consequences (Basak and Sathyanarayana, 2010; Al-Worafi, 2014). People depend on pharmacies for seeking healthcare due to flexible opening hours, shorter waiting time, convenience of access, and low price of medicines (Kafle et al., 1992; Saha and Hossain, 2017). According to the World Health Organization (WHO), globally there are almost 2.6 million pharmacists and pharmaceutical personnel deployed at retail pharmacies whose roles are important for promoting good pharmacy practices with the patients (Munna and Islam, 2019). In developing countries, drug dispensers deployed at pharmacies are considered to be the first contact point of a patient and any kind of drugs can be purchased over the counter without a prescription (Choudhury, 2016). Further, research studies conducted in South Asia found that medicines prescribed, dispensed or sold at the pharmacies are often inappropriate or inadequate leading to irrational use of medicines (Patel et al., 2005; Toklu et al., 2009; Toklu et al., 2010; Chaturvedi et al., 2012; Benkhaial et al., 2019; Nepal et al., 2021).

In Bangladesh, Graduate Pharmacists are labelled as Grade A pharmacists and those who complete a 3-year Diploma in Pharmacy course are labelled as Grade B pharmacists. Both Grade A and Grade B pharmacists are eligible for obtaining pharmacy license and opening medicine shops (PCB, 2023a). However, due to shortage of qualified Grade A and Grade B pharmacists, the government has introduced a new cadre of pharmacy professionals known as Grade C pharmacists who required to have a Secondary School Certificate (10 years of schooling) and a “Grade C pharmacist certificate” by completing a 3-month training course jointly conducted by the Pharmacy Council of Bangladesh (PCB) and Bangladesh Chemist and Druggist Samity (BCDS) (an organization of drug dispensers) in order to become eligible for applying to the Directorate General of Drug Administration (DGDA) for a pharmacy license (Mazid and Rashid, 2011; Bates et al., 2016; DGDA, 2016; Begum et al., 2021).

The Pharmacy Council, is a government regulatory body responsible for regulating education, training, and registration of Grade A, Grade B and Grade C pharmacists in Bangladesh (PCB, 2023b). According to DGDA, there are 106,919 licensed retail medicine shops in Bangladesh and a similar number of unlicensed retail medicine shops are located countrywide (DGDA, 2017). The licensed retail medicine shops are generally located in marketplaces run by Grade C pharmacists who are authorized to dispense both over-the-counter (OTC) and prescription medicines (Begum et al., 2021).

The public-private partnership launched to improve access to quality medicines and pharmaceutical services in rural Tanzania has demonstrated good dispensing practices by the drug dispensers at private pharmacies (Kagashe et al., 2011). Studies conducted in Bangladesh have documented that training and education improved drug dispensing behavior of drug dispensers and availability of trained drug dispensers at the pharmacies (Saha et al., 2017; Mondal et al., 2021). Another study has documented that drug dispensers in Bangladesh who received training had also demonstrated better knowledge about antihypertensive and anticonvulsant medications, and less frequently dispensing antibiotics for uncomplicated acute respiratory illnesses in children (Chowdhury et al., 2018).

In 2015, DGDA in Bangladesh launched an accreditation initiative with the technical support of Management Sciences for Health (MSH) and other stakeholders in order to develop the first standards for retail medicine outlets. The goal was to reduce inappropriate use of medicines in the country by introducing two new levels of medicine outlets—Model Pharmacy (level 1) and Model Medicine Shop (level-2). A Model Pharmacy (level 1) is served, managed and supervised by a graduate pharmacist (Grade A) and a Model Medicine Shop (level 2) is operated by a Grade C pharmacist at a minimum.

A standard guideline entitled “Establishment and Operations of Model Pharmacy and Model Medicine Shop in Bangladesh” was also developed. Under this accreditation program, a total of 5,059 Grade C pharmacists were trained on the guideline of Model Pharmacy and Model Medicine Shop under the Better Health in Bangladesh (BHB) project between 2018 and 2020 in order to improve Good Pharmacy Practice (GPP). Management Sciences for Health (MSH) supported the DGDA to facilitate the GPP training that included minimum requirements of premises of model medicine shop (permanent structure, leakage free roof and ceiling), medicine storage system, maintaining temperature, dispensing of over the counter (OTC) drugs, patient counseling and labeling of medicines. There is scarcity of evidence whether the GPP training offered by MSH led to improved services delivered by the trained Grade C pharmacists deployed at the model medicine shops compared to the Grade C pharmacists who did not have the same training and deployed at the regular medicine shops. We assessed the knowledge of GPP and observed general dispensing practices between the trained and the non-trained Grade C pharmacists, and identified challenges for achieving GPP in Bangladesh.

## 2 Material and method

### 2.1 Study site and recruitment of study participants

We conducted a cross sectional survey in 11 districts in Bangladesh among a sub sample of 5,059 Grade C pharmacists who have received GPP training provided by MSH between January 2018 and June 2020. We estimated the sample size based on a study conducted in Bangladesh, which has reported that knowledge of dispensing was 11% higher about side effects of drugs, 12% higher about counseling patients about administration of medicine, and 14% higher about the difference between OTC drugs and prescription drugs among the trained Grade C pharmacists deployed at model pharmacies compared to the non-trained Grade C pharmacists deployed at retail medicine shops. Considering the difference in knowledge ranging from 10% to 14% between the trained and the non-trained Grade C pharmacists, we calculated that the largest sample size would be required to detect a 12% knowledge gap between the trained and the non-trained Grade C pharmacists, and we would need to recruit 220 participants in each group considering 95% level of significance, 90% power, and a 5% non-response rate.

We created a database of trained Grade C pharmacists in each district using the list provided by MSH and randomly recruited 220 Grade C pharmacists. The total number of trained Grade C pharmacists to be recruited in each district was determined by using proportional allocation technique to ensure representation of the trained Grade C pharmacists in each district (Supplementary Table S1). We created another database of non-trained Grade C pharmacists deployed at a drug outlet located within a 1-km radius of the recruited trained Grade C pharmacists and randomly selected one non-trained Grade C pharmacist from the list against a trained Grade C pharmacist. Any participant who could not show a “Grade C pharmacist certificate” or refused to provide a written consent for an interview or an observation was excluded from the study.

### 2.2 Data collection

A group of trained researchers first made a direct observation of the dispensing practices of each study participant and documented findings using a structured observation tool (checklist). Observation of dispensing practices included dispensing of prescription-only-medicine without prescription, temperature maintenance at the medicine shop, medicine storage system, counseling given to the customers and labeling medicines at the time of dispensing. The team also observed the surroundings of the medicine shops following a standard guideline approved by DGDA to assess the minimum requirements of premises of a medicine shop, ‘no smoking’ sign displayed, and having a functional air conditioner inside the pharmacy to maintain the ambient temperature. The checklist was filled up immediately after observation and the duration of observation varied depending on the setting or situation of the pharmacy at the time of observation.

Data were collected using a pretested structured questionnaire, which was developed including the tools used by MSH for providing training under BHB project. Face to face interviews were conducted to collect data on socio-demographic status, economic status, types of training, number of the training, duration of the training course, knowledge on general dispensing behavior, including, premises of the medicine shop, medicine storage system, temperature maintenance for the medicine shop, patient counseling and labeling of the medicine. Challenges faced by the dispensers at work and suggested solutions were also explored.

### 2.3 Data analysis

Descriptive statistics were applied to describe the characteristics of the study participants including age, sex, education, monthly income, and training received. Bivariate analysis was conducted to examine any differences in socio-demographic characteristics, knowledge of dispensing behavior, and observed dispensing practices between the trained and the non-trained Grade C pharmacists. Chi-square test was applied for comparing proportion, independent sample t-test was applied for comparing mean and two-sample median test was applied for comparing median differences between the two groups. Logistic regression model was applied to estimate odds ratio in order to explore association of knowledge and practice between the two groups. Statistical Package for the Social Sciences (SPSS) software version 21 was used for data analysis and statistical significance was set at a threshold of *p* < 0.05. (Corp, 2016).

### 2.4 Ethics

The study obtained approval from the Ethical Review Committee of International Centre for Diarrhoeal Disease Research, Bangladesh (icddr,b) and Bangladesh Medical Research Council (BMRC). Written informed consent was obtained from each study participant prior to direct observation and conducting interview.

## 3 Results

### 3.1 Socio-demographic characteristics

Between February and March 2021, the study enrolled 440 Grade C pharmacists (220 trained, and 220 non-trained), including 80 from Dhaka (18.0%), 36 from Narshindhi (8.0%), 54 from Chattagram (12.0%), 56 from Chandpur (12.5%), 32 from Maulovibazar (7.0%), 30 from Mymensingh (7.0%), 24 from Natore (6.0%), 36 from Rangpur (8.0%), 52 from Khulna (12.0%), 14 from Bagherhat (3.0%) and 26 from Jhalokathi (6.0%). The mean age of the participants was 41 years (SD ± 10.5) and 98.4% were male. About a third (32.3%) have completed a higher secondary school certificate education (12 years of schooling) and the average monthly income was 238.8 USD (Range: 179.1–417.9 USD). Age, education and monthly income did not vary between the trained and the non-trained pharmacists (Table 1).

**TABLE 1:** Respondents’ demographic and professional characteristics (N = 440).

Variables	Total (N = 440)	Trained Grade C pharmacists (N = 220)	Non-trained Grade C pharmacists (N = 220)	*p*-value
Sex, n (%)				
Male	433 (98.4)	216 (98.2)	217 (98.6)	0.703
Female	7 (1.6)	4 (1.8)	3 (1.4)	
Age (year)± SD	40.58 ± 10.54	41.48 ± 10.879	39.46 ± 9.964	0.145
Education, n (%)				
Completed SSC^1^	118 (26.8)	61 (25.7)	57 (25.5)	0.330
Completed HSC^2^	142 (32.3)	77 (35.8)	65 (30.2)	
Completed Graduation^3^	103 (23.4)	50 (24.3)	53 (22.2)	
Post graduate^4^	77 (17.5)	32 (14.2)	45 (21.7)	
Monthly income from the pharmacy profession, Median (25th −75th Percentile)	238.80 (179.1–417.9) USD	250.74 (179.1–477.6) USD	238.80 (179.1–358.2) USD	0.408
Working hour per day (hr.) ±SD	10.9 ± 2.5 h	10.6 ± 2.3 h	11.2 ± 2.6 h	0.031
Working days per week± SD	6.81 ± 0.482 days	6.81 ± 0.391 days	6.81 ± 0.563 days	0.989
Working experience in the selected pharmacy (years)± SD	10.48 ± 8.9	12.22 ± 9.2	8.74 ± 8.2	0.001
Time spent per customer to dispense medicine (min)± SD	9.84 ± 4.770 min	10.20 ± 5.01 Min	9.47 ± 4.49 Min	0.045
Training received other than the training provided by BHB (N = 440)	126 (28.6%)	67 (30.5%)	59 (26.8%)	<0.001
Number of the training received (N = 126)	**N =** 126 **(%)**	**N =** 67 **(%)**	**N =** 59 **(%)**	
1 training received	59 (46.8)	30 (45.3)	29 (49.2)	0.736
2 training received	34 (27.3)	18 (27.4)	16 (27.1)	
3 training received	21 (16.2)	12 (17.9)	9 (13.6)	
4 training received	4 (3.2)	2 (2.1)	2 (5.1)	
≥5 training received	8 (6.5)	5 (7.4)	3 (5.1)	
When last training was received	**N =** 126 **(%)**	**N =** 67 **(%)**	**N =** 59 **(%)**	
<1 year	11 (8.9)	6 (9)	5 (8.5)	**<0.001**
1to <2 years	65 (51.9)	42 (61.8)	23 (18.6)	
2 to <3 years	30 (24)	15 (23.1)	15 (27.1)	
3 to <4 years	8 (5.8)	2 (3.5)	6 (13.6)	
4 years and above	12 (9.3)	2 (2.5)	10 (32.2)	

^1^ Secondary School Certificate/Madrasha education board (Dakhil)

^2^Higher Secondary certificate//Madrasha education board (Alim)

^3^BA (pass course)/Honors/Fazil

^4^(Masters/Kamil).

### 3.2 Work experience

The average duration of work experience of the participants in the respective medicine shop was 10 years and, the trained Grade C pharmacists had longer work experience than the non-trained (12 years vs 8 years, *p* < 0.001). The Grade C pharmacists reported to be on duty almost every day in a week (7 days ± .05) and the mean dispensing time was 10.9 h (SD ± 2.5) per day with a longer hour among the non-trained than the trained Grade C pharmacists (11.2 ± 2.6 vs 10.6 ± 2.3; *p* = 0.03). The average dispensing time per customer was 9 min (SD 9.84 ± 4.770) and there was no difference between the two groups (Table 1).

### 3.3 Training received

Overall, 28.6% of the participants have received additional training beyond Grade C pharmacist certificate (other than the GPP training offered by MSH) with a higher proportion among the trained than the non-trained pharmacists (30.5% vs 26.8%, *p* < 0.001). About half of the respondents reported to have received at least one additional training (46.8%) and 6.5% reported receiving at least 5 training since obtained the certificate; although there was no difference between the two groups in terms of the number of training received. Among them 51.9% mentioned receiving a training within 2 years and among them the proportion of the trained Grade C pharmacists was higher than the non-trained (61.8% Vs. 18.6%; *p* < 0.001). (Table 1).

### 3.4 Knowledge on guidelines of dispensing medicines

A total of 95.7% participants reported to have the knowledge about a guideline for dispensing medicines with a higher proportion reported among the trained than the non-trained (Table 2). Almost all participants in both groups reported to have the knowledge that prescription-only medicines should not be sold without a prescription (99%), expired medicine should not be dispensed (99.77%), only registered medicine should be dispensed (99.77%), no medicines should be dispensed directly to a child younger than 12 years of age (98.18%), and no difference was observed between the two groups (Table 2).

**TABLE 2:** Knowledge of dispensing behavior of the trained and non-trained Grade C technicians.

	Knowledge of the dispensing behavior of grade C technicians				
	Total N = 440 (%)		Trained Grade C pharmacists N = 220 (%)	Non-trained Grade C pharmacists N = 220 (%)	*p*-value
Knowledge about availability of guidelines for dispensing medicine	421 (95.7%)		217 (98.6%)	204 (92.7%)	**0.002**
Knowledge about the guideline of dispensing medicine	412 (93.6%)		215 (97.72%)	197 (89.54%)	0.001^a^
Opinion regarding the following statement of dispensing medicine					
Prescription only medicines should not be dispensed without prescription	Right	438 (99)	220 (100)	218 (99)	0.156
	Wrong	2 (1)	-	2 (0.917)	
	Don’t know	-	-	-	
Physician samples cannot be dispensed	Right	433 (98.4)	217 (98.18)	216 (98.63)	0.135
	Wrong	3 (0.7)	-	3 (1.36)	
	Don’t know	4 (0.9)	3 (1.36)	1 (0.45)	
Expired medicine cannot be dispensed	Right	439 (99.77)	219 (99.54)	220 (100)	
	Wrong	1 (0.3)	1 (0.45)	-	
	Don’t know	-	-	-	
All medicines dispensed must be registered by DGDA	Right	439 (99.77)	220 (100)	219 (99)	0.317
	Wrong	1 (0.3)	-	1 (0.45)	
	Don’t know	-	-	-	
Tablet and capsule should be dispensed using at the appropriate tool (i.e., counting tray)	Right	335 (76.13)	160 (72.7)	175 (8)	0.184
	Wrong	37 (8.4)	23 (10.5)	14 (6.4)	
	Don’t know	68 (15.5)	37 (16.8)	31 (14.1)	
No medicines should be dispensed directly under 12 years old children	Right	432 (98.18)	215 (97.7)	217 (98.6)	0.097
	Wrong	2 (0.5)	-	2 (.9)	
	Don’t know	6 (1.4)	5 (2.3)	1 (.5)	
Knowledge about the guideline of the model medicine shop (premises)		269 (61.13)	205 (93.18)	64 (29.09)	0.001^a^
Roof and ceiling of model medicine shop should be leakage free		204 (46.4)	152 (69.1)	52 (23.6)	0.001^a^
Structure of model medicine shop should be Permanent		273 (62.04)	191 (86.81)	82 (37.3)	0.001^a^
The floor of model medicine shop should be Permanent (Smooth)		312 (70.90)	207 (94.09)	105 (47.72)	0.001^a^
The wall of model medicine shop should be permanent (Smooth)		309 (70.22)	207 (94.09)	102 (46.36)	0.001^a^
Potable water supply should be in a model medicine shop		298 (67.62)	197 (89.5)	101 (45.9)	0.001^a^
Medicine shop should be maintained to minimize the entry of animal, rodent etc.		185 (42.04)	139 (63.18)	46 (20.90)	0.001^a^
Knowledge of the temperature of model medicine shop should be controlled		302 (68.63)	202 (91.81)	100 (45.45)	0.001^a^
AC is needed to control room temperature		209 (47.5)	132 (60.0)	77 (35.0)	0.001^a^
Fan is needed to control room temperature		201 (45.7)	135 (61.4)	66 (30.0)	0.001^a^
Thermometer is needed to maintain room temperature		252 (57.3)	187 (85.0)	65 (29.5)	0.001^a^
Electricity and backup power supply is needed (generator, IPS or solar panel)		84 (19.1)	57 (25.9)	27 (12.3)	0.001^a^
Knowledge about the medicine storage system		292 (66.4)	203 (92.3)	89 (40.5)	0.001^a^
The knowledge about the way of medicine storage system of the model medicine shop					
The medicine should be stored to the indicated temperature		192 (43.6)	128 (58.2)	64 (29.1)	0.001^a^
OTC medicine and prescription-only medicine should be stored separately		259 (58.9)	183 (83.2)	76 (34.5)	0.001^a^
Pharmaceutical and non-pharmaceutical products should be stored separately		199 (45.2)	143 (65.0)	56 (25.5)	0.001^a^
Label should be attached on different shelves		174 (39.5)	129 (58.6)	45 (20.5)	0.001^a^
Unani, Ayurvedic, herbal medicine should be stored separately on different shelves		158 (35.9)	112 (49.1)	46 (79.1)	0.001^a^
Knowledge about the patient counseling on		422 (95.9)	219 (99.5)	203 (92.3)	0.001
Type of medicine		133 (30.22)	75 (34.09)	58 (26.36)	
Dosage of the medicine		371 (84.31)	188 (85.45)	183 (83.18)	0.512
Purpose		168 (38.18)	88 (40)	80 (36.36)	0.432
Instruction on drug interaction, food interaction		124 (28.18)	69 (31.36)	55 (25)	0.138
Side effect		157 (35.68)	84 (38.18)	73 (33.18)	0.274
Duration of the course of treatment		306 (69.54)	172 (78.18)	134 (60.90)	0.001^a^
Usage pattern of drug		357 (81.13)	183 (83.18)	174 (79.09)	0.273
Expiration date		190 (43.18)	99 (45)	91 (41.36)	0.441
Storage system		87 (19.77)	52 (23.63)	35 (15.90)	0.042^a^
Knowledge of labeling of the medicine					
Labeling of dispensed medicine is clear in local language		375 (85.2)	200 (90.9)	175 (79.54)	0.001^a^
Level of the container must be indicated (patient name and address, name of the medicine, direction for use, expiry date)		337 (76.6)	180 (81.8)	157 (71.36)	0.010^a^

^a^ Statistical significance at *p* < 0.05.

### 3.5 Knowledge about the minimum requirements of premises

More than half of the participants reported to have the knowledge of minimum requirements of premises of a model medicine shop (61.13%). However, the trained Grade C pharmacists had more knowledge about maintaining leakage free roof and ceiling (69.1% vs 23.6%, *p* < 0.001), having a permanent structure (86.81% vs 37.3%, *p* < 0.001), supply of potable water (89.5% vs 45.9%, *p* < 0.001) and need of a room thermometer to monitor the room temperature (85.0% vs 29.5%, *p* < 0.001) than the non-trained Grade C pharmacists (Table 2).

### 3.6 Knowledge about medicine storage systems

About two-third (66.4%) of the participants were aware about medicine storage systems with a higher proportion reported among the trained than the non-trained (92.3% vs 40.5%, *p* < 0.001). However, knowledge about specifics of medicine storage system was much better among the trained than the non-trained pharmacists in terms of medicines should be stored at the indicated temperature (58.2% vs 29.1%), OTC medicines and prescription-only medicines should be stored separately (83.2% vs 34.5%), pharmaceutical and non-pharmaceutical products should be stored separately (65.0% vs 25.5%), and label should be attached on different shelves (58.6% vs 20.5%, *p* = 0.001) (Table 2).

### 3.7 Knowledge about counseling the customers and labeling medicines

The trained Grade C pharmacists had better knowledge about counseling the customers while dispensing medicine (99.5% vs 92.3%, *p* < 0.001) more frequently mentioned that labeling should be in local language (91% vs 80%, *p* < .001). Trained Grade C pharmacists also had a better knowledge about labeling the medicine container with patient name, address, and medicine name than the non-trained Grade C pharmacists (81.8% vs 71.36%, *p* = 0.01) (Table 2).

### 3.8 Findings of the direct observation of dispensing behavior

Overall, half of the Grade C pharmacists (49.8%) were observed to have dispensed medicines without a prescription, and it was more frequently observed among the non-trained than the trained (53.6% vs 45.9%, *p* = 0.01) (Table 3). Most of the participants used a fan for maintaining ambiant temperature (97.3%) and use of a room thermometer was more frequently observed among the trained than the non-trained (56.8% vs 26.8%, *p* = 0.001) (Table 3). About two-third of the participants were found to store pharmaceutical and non-pharmaceutical products separately (68.9%). The trained Grade C pharmacists were more frequently observed to attach a label on different selves (61.4% vs 24.1%; *p* = 0.001) and store Unani, Ayurvedic and Herbal medicines separately on different shelves (73.6% vs 61.4%) than the non-trained (Table 3).

**TABLE 3:** Observation of the dispensing practice of trained and non-trained Grade C technicians.

Variables	Total N = 440 (%)	Trained Grade C pharmacists N = 220 (%)	Non-trained Grade C pharmacists N = 220 (%)	*p*-Value
Dispensing of prescription only medicines without prescription	219 (49.8)	101 (45.9)	118 (53.6)	0.01
Premises of the pharmacies				
Roof of the shop leakage free	419 (95.2)	212 (96.4)	207 (94.1)	0.25
Permanent structure	411 (93.6)	208 (95.0)	203 (92.3)	0.25
Smooth floor	328 (74.5)	168 (76.4)	160 (72.7)	0.38
Potable water available	247 (56.1)	130 (59.1)	117 (53.2)	0.21
Animal, rodent cannot enter easily	315 (71.6)	151 (68.6)	164 (74.5)	0.17
Equipment available at the medicine shop to maintain room temperature				
AC	16 (3.6)	10 (4.5)	6 (2.7)	0.31
Fan	428 (97.3)	217 (98.6)	211 (95.9)	0.08
Thermometer to maintain room temperature	184 (41.8)	125 (56.8)	59 (26.8)	0.001^a^
Electricity and backup power supply (generator, IPS or solar panel)	185 (42.0)	100 (45.5)	85 (38.6)	0.15
Medicine storage system in the medicine shop				
Refrigerator available	262 (59.8)	136 (62.1)	126 (57.5)	0.33
Pharmaceutical and non-pharmaceutical products stored separately	303 (68.9)	159 (72.3)	144 (65.5)	0.12
Label attached on different shelves	188 (42.7)	135 (61.4)	53 (24.1)	0.001^a^
Unani, Ayurvedic, herbal medicine stored separately on different shelves	297 (67.5)	162 (73.6)	135 (61.4)	0.006^a^
Topics of the counseling				
Type of medicine	178 (40.5)	100 (45.5)	78 (35.5)	0.03^a^
Dosage of the medicine	306 (69.5)	157 (71.4)	149 (67.7)	0.40
Purpose	149 (33.9)	86 (39.1)	63 (28.6)	0.02
Instruction on drug interaction, food interaction	106 (24.2)	63 (28.6)	43 (19.7)	0.03
Side effect	145 (33)	81 (36.8)	64 (29.2)	0.09
Duration of the course of treatment	302 (68.8)	160 (72.7)	142 (64.8)	0.001^a^
Labeling of the medicine				
Labeling of dispensed medicine is clear in local language	256 (58.3)	139 (63.2)	117 (53.4)	0.038
Label of the container must be indicated (patient name and address, name of the medicine, direction for use, expiry date)	130 (29.6)	77 (35.0)	53 (24.2)	0.013

^a^ Statistical significance at *p* < 0.05.

The trained Grade C pharmacists were more frequently bserved to counsel the customers on the type of medicine (45.5% vs 35.5%), purpose of taking medicine (39.1% Vs 28.6%), interaction with drug or food (28.6% vs 19.6%) and duration of the course of the medicine (72.7% vs 64.8%) compared to the non-trained (*p* < 0.05). Trained Grade C pharmacists were also found to label the medicines in local language (63.2% vs 53.4%; *p* = 0.038) and indicated patient name, address, name of medicine, direction for use and expiry date while labeling the container (35.0% vs 24.2%; *p* = 0.013) than the non-trained (Table 3).

### 3.9 Challenges of pharmacy practices

Overall, 39.5% technicians reported bad behavior of customers or lack of attention to listen instruction of dispensing medicines followed by lack of economic incentives (31.1%), lack of knowledge of medicines and training (28.6%), lack of communication skill with patients (12.5%), financial problem of customer (12.3%), poor salary (9.1%) and not getting enough respect from customers (5.6%) (Table 4).

**TABLE 4:** Challenges and potential solution.

Challenges or problems are faced by the dispensers	Total N = 440 (%)	N = 220 (%)	N = 220 (%)	*p*-Value
Bad behaviour of the customers (unwilling to purchase the full course of medicines or forcefully purchased medicines without prescriptions)	174 (39.54)	83 (37.72)	91 (41.36)	0.393
Lack of economic incentives	137 (31.13)	70 (31.81)	67 (30.45)	0.709
Lack of knowledge of medicine and training	126 (28.63)	68 (30.90)	58 (26.36)	0.315
Inadequate supplies of the medicine	98 (22.27)	48 (21.81)	50 (27.72)	0.839
Financial problem of customer	54 (12.27)	27 (12.27)	27 (12.27)	0.100
Lack of communication skills with the patients	55 (12.5)	34 (15.45)	21 (9.54)	0.058
Poor salary	40 (9.09)	21 (9.5)	19 (8.63)	0.729
Not getting enough respect	25 (5.6)	13 (5.9)	12 (5.45)	0.828
**Potential solutions to overcome challenges of dispensers**				
Arrangement of regular training	245 (55.7)	128 (58.2)	117 (53.2)	0.208
Providing incentive or financial facilities	160 (36.4)	78 (34.09)	82 (37.3)	0.616
Improvement of the salary structure	64 (14.54)	32 (14.54)	32 (14.54)	0.968
Implementing a monitoring system by the government	35 (7.95)	18 (8.18)	17 (7.27)	0.749

### 3.10 Potential solutions of the challenges

More than half of the Grade C pharmacists (55.7%) reported that arranging GPP training on a regular basis is very important for improving the quality of pharmacy practices. One-third of them mentioned that introducing a provision of incentive or financial support would be helpful for achieving GPP (36.4%), while a smaller proportion mentioned a better salary structure (14.5%) and implementing a monitoring system by the government (7.9%) would be imperative for overcoming the challenges. No difference was observed in reporting of the potential solutions between the trained and the non-trained Grade C pharmacists (Table 4).

### 3.11 Association between knowledge and observed practices between the trained and non-trained

Generally, the association of knowledge and practices among the trained Grade C pharmacists was higher than the non-trained in terms of premises of the pharmacies across most of the parameters assessed, including permanent structure (OR: 12.47, 95% CI: 11.96–12.97), smooth floor (OR: 17.23, 95% CI: 16.49–17.97), potable water availability (OR: 24.52, 95% CI: 23.66–25.37), prevention of easy entry for animals and rodents (OR: 6.68, 95% CI: 6.24–7.12), and designated waiting place for customers (OR: 3.73, 95% CI: 3.33–4.13). Association of knowledge and practice in terms of utilizing a thermometer to regulate the room temperature was higher among the trained than the non-trained (OR: 5.21, 95% CI: 3.99–6.42), although association between the two groups in terms of need of an air conditioner or fan or back up power supply was similar.

A higher association between knowledge and practice was observed among the trained Grade C pharmacists in terms of separating pharmacological and non-pharmacological products (OR: 1.87, 95% CI: 1.23–2.51) and attaching labels on different shelves (OR: 2.1, 95% CI: 1.51–2.7), although no difference was observed in terms of storing Unani/Ayurvedic/herbal medicines on different shelves. Association of knowledge and practice in terms of counseling on the duration of course of treatment was better among the trained than the non-trained (OR: 1.88, 95% CI: 1.34–2.43), no difference was observed in terms of counseling on types, dosage, purpose, drug or food interaction, and side effect of medicines (Supplementary Table S2). Overall, the association between knowledge and practice for labeling of medicines was not different between the two groups.

## 4 Discussion

This study has demonstrated that the trained Grade C pharmacists had better knowledge about good pharmacy practices including counseling the customers, which is similar to the findings in Tanzania that has demonstrated that trained drug dispensers had better knowledge on antimicrobial resistance (AMR) and were better aware about giving instruction while dispensing medicine to customers (Valimba et al., 2014). Our study findings also support a previous evidence in Bangladesh documenting that trained drug dispensers were likely to have correct knowledge on anti-hypertensive and anticonvulsant drugs than those who had no training (Valimba et al., 2014; Roy et al., 2020).

Our study has further confirmed from observation that the trained Grade C pharmacists had better medicine dispensing practices, correct labeling of medicines and less frequent dispensing of medicines without prescription than the non-trained Grade C pharmacists, which are similar to the previous studies in Tanzania and Bangladesh indicating that training is a very important tool for improving knowledge of correct dispensing of medicines by Grade C pharmacists in Bangladesh (Valimba et al., 2014; Chowdhury et al., 2018; Begum et al., 2021).

Our study has demonstrated that the trained Grade C pharmacists more frequently counseled the customers than the non-trained, which is aligned with other studies conducted in LMICs, such as, a positive impact of training has been demonstrated in Pakistan on counseling the customers about medicine dosage, completing the full course, storage conditions of the drug etc (Naveed et al., 2014; Dos Reis et al., 2019).

In this study, the Grade C pharmacists reported encountering customers with bad behavior. These barriers that impeded dispensing drugs according to the guidelines used for pharmaceutical management (MSH, 2023). These challenges can be mitigated by reinforcement of laws and regulation through implementation of a strong monitoring system by the government, which have been documented in this study. However, some factors influence drug dispensing behavior like training, knowledge, professional compensation or financial facilities, better salary structure, government aid etc. and have been suggested by MSH as well (MSH, 2023).

This study had a few limitations. First our selection of the participants was limited to the Grade C pharmacists who were trained by the MSH on GPP, hence our sample may not represent Grade C pharmacists who received similar training offered by accredited organizations other than the MSH. Second, the number of the Grade C pharmacists who were trained by the MSH was not proportionally distributed across different administrative divisions, and were limited to only 11 districts, hence we cannot claim that our results generalizable for Grade C pharmacists in Bangladesh.

However, our goal was to assess the impact of the GPP training provided by the MSH and we have randomly selected the participants from the full list of trained Grade C pharmacists, hence our study population was representative of the cohort participated in the GPP training offered by the MSH. Further we have acquired reasonably a large sample size of the GPP trained pharmacy technicians among both of the trained and the non-trained Grade C pharmacists for conducting the surveys and a direct observation was carried out on every participant in the survey. As such, this research has generated a credible evidence of the medicine dispensing practices among the trained Grade C pharmacists in comparison with the non-trained who were recruited from the same working environment as of the trained Grade C pharmacists. Third, we used a guideline of model pharmacy and model medicine shop to gather data from the respondents, which has added strength to the study on top of a robust study design adopted by the study. The Grade C pharmacists received 2 weeks of training between 2018 and 2020 in 11 districts of Bangladesh, and our evaluation conducted in 2021 has demonstrated that GPP training had positive impact on knowledge and practices among the trained Grade C pharmacists beyond six month of GPP training, indicating long term impact.

Overall, our study findings suggest that GPP training has generally enhanced skills of the Grade C pharmacists in terms of dispensing medicine, particularly avoiding selling drugs without prescriptions of physicians, completing full course of medicines, maintaining proper dosage, storing medicine in appropriate temperature, which could have rendered beneficial effect on treatment outcome of the patients who received medicines from the trained Grade C pharmacists. Although the study did not have the scopes to review patient benefit due to GPP training of the Grade C technicians, our study has supported that there has been potential improvement in medicine dispensing practices among drug dispensers following GPP training, which support the value of GPP in a setting with limited number of graduate pharmacists, such as, Bangladesh. Overall, training helps Grade C pharmacists in upscaling their skills for dispensing medicine, which potentially would impact on patient outcome indirectly. Therefore, the findings of the study have implications for periodic conduct of training among drug dispensers for promoting good pharmacy practices in Bangladesh and other LMICs.

## 5 Conclusion

The training offered to Grade C pharmacists by the MSH under the BHB project led to better knowledge on GPP and dispensing practices in Bangladesh. Implementation of training among drug dispensers and a proper monitoring system applied by the government would support implementing GPP in Bangladesh.

## Data Availability

The original contributions presented in the study are included in the article/Supplementary Materials, further inquiries can be directed to the corresponding author.
