# Supplementary material for: Assessment of the impact of good pharmacy practices training among drug dispensers in Bangladesh
Source: Front Pharmacol. 2023 Jul 12;14:1139632. doi: 10.3389/fphar.2023.1139632 (PMC10370418; doi:10.3389/fphar.2023.1139632)
Supplement: Supplementary file 1 [file DataSheet1.docx]

**Supplementary file**

Table S1. Estimated sample size based on proportional allocation procedure to 11 districts

| **Districts** | **Total trained technicians** | **Proportional allocation of sample size**  **(Trained technicians=220** | | **Proportional allocation of sample size**  **(Non-trained technicians=220** | |
| --- | --- | --- | --- | --- | --- |
| Dhaka | 883 | 40 | 18% | 40 | 18% |
| Chandpur | 643 | 28 | 13% | 28 | 13% |
| Chattagram | 642 | 29 | 13% | 29 | 13% |
| Khulna | 574 | 25 | 11% | 25 | 11% |
| Narshindhi | 404 | 18 | 8% | 18 | 8% |
| Rangpur | 413 | 18 | 8% | 18 | 8% |
| Moulavi bazaar | 370 | 16 | 7% | 16 | 7% |
| Mymensing | 324 | 14 | 6% | 14 | 6% |
| Nator | 244 | 11 | 5% | 11 | 5% |
| Jhalokathi | 280 | 13 | 6% | 13 | 6% |
| Bagherhat | 160 | 8 | 4% | 8 | 4% |
| **Total** | **4937** | **220** | **100%** | **220** | **100%** |

**Table S2.** Association between knowledge and practices of model medicine shop among trained and non-trained practitioners

|  | Trained [Total: 220 (%)] | | | Non-trained [Total: 220 (%)] | | | Odds ratio [Trained vs. non-trained] |
| --- | --- | --- | --- | --- | --- | --- | --- |
|  | Practices | | Trained -Odds | Practices | | Nontrained- odds |  |
|  | With Knowledge | Without knowledge |  | With Knowledge | Without knowledge |  |  |
| Premises of the pharmacies |  |  |  |  |  |  |  |
| Roof of the shop leakage free | 144 | 68 | 2.1 | 160 | 47 | 3.4 | 0.62 (0.19-1.06) |
| Permanent structure | 182 | 26 | 7.0 | 73 | 130 | 0.6 | **12.47 (11.96-12.97)^*^** |
| Smooth floor | 159 | 9 | 17.7 | 81 | 79 | 1.0 | **17.23 (16.49-17.97)^*^** |
| Potable water available | 123 | 7 | 17.6 | 43 | 60 | 0.7 | **24.52 (23.66-25.37)^*^** |
| Animal, rodent cannot enter easily | 132 | 80 | 1.7 | 41 | 166 | 0.2 | **6.68 (6.24-7.12)^*^** |
| Designated waiting place for customers | 144 | 72 | 2.0 | 74 | 138 | 0.5 | **3.73 (3.33-4.13)^*^** |
| Temperature of model medicine shop |  |  |  |  |  |  |  |
| AC is needed to control room temperature | 10 | 0 |  | 4 | 0 |  |  |
| Fan is needed to control room temperature | 133 | 77 | 1.7 | 66 | 33 | 2.0 | 0.86 (0.36-1.37) |
| Thermometer is needed to maintain room temperature | 119 | 5 | 23.8 | 32 | 7 | 4.6 | **5.21 (3.99-6.42)^*^** |
| Electricity and backup power supply is needed (generator, IPS or solar panel) | 38 | 56 | 0.7 | 16 | 29 | 0.6 | 1.23 (0.49-1.97) |
| Medicine storage system in the medicine shop |  |  |  |  |  |  |  |
| Pharmaceutical and non-pharmaceutical products stored separately | 144 | 35 | 4.1 | 44 | 20 | 2.2 | **1.87 (1.23-2.51)^*^** |
| Label attached on different shelves | 101 | 48 | 2.1 | 32 | 32 | 1.0 | **2.1 (1.51-2.7)^*^** |
| Unani, Ayurvedic, herbal medicine stored separately on different shelves | 88 | 61 | 1.4 | 37 | 27 | 1.4 | 1.05 (0.46-1.65) |
| Topics of the counseling |  |  |  |  |  |  |  |
| Type of medicine | 48 | 52 | 0.9 | 40 | 36 | 1.1 | 0.83 (0.23-1.43) |
| Dosage of the medicine | 146 | 11 | 13.3 | 134 | 10 | 13.4 | 0.99 (0.1-1.88) |
| Purpose of usage of medicine | 53 | 33 | 1.6 | 43 | 20 | 2.2 | 0.75 (0.06-1.43) |
| Instruction on drug interaction, food interaction | 41 | 22 | 1.9 | 30 | 13 | 2.3 | 0.81 (0.34-1.64) |
| Side effect | 52 | 29 | 1.8 | 48 | 16 | 3.0 | 0.6 (0.13-1.32) |
| Duration course of the treatment | 131 | 29 | 4.5 | 96 | 40 | 2.4 | **1.88 (1.34-2.43)^*^** |
| Labeling of the medicine |  |  |  |  |  |  |  |
| Labeling of dispensed medicine is clear in local language | 131 | 3 | 43.7 | 110 | 1 | 110.0 | 0.4 (0.88-2.67) |
| Label of the container must be indicated (patient name and address, name of the medicine, direction for use, expiry date) | 74 | 1 | 74.0 | 50 | 1 | 50.0 | 1.48 (0.31-4.27) |

^$^ Chi-square test of independence ^*^ Statistically significant at P<0.05
